# COMPARISON OF METAL ARTEFACTS FOR DIFFERENT DUAL ENERGY CT TECHNIQUES

**DOI:** 10.1093/rpd/ncab105

**Published:** 2021-08-04

**Authors:** E Pettersson, A Bäck, A Thilander-Klang

**Affiliations:** Department of Radiation Physics, Institute of Clinical Sciences, Sahlgrenska Academy, University of Gothenburg, Gothenburg SE-413 45 Sweden; Department of Therapeutic Radiation Physics, Medical Physics and Biomedical Engineering, Sahlgrenska University Hospital, Gothenburg SE-413 45, Sweden; Department of Radiation Physics, Institute of Clinical Sciences, Sahlgrenska Academy, University of Gothenburg, Gothenburg SE-413 45 Sweden; Department of Therapeutic Radiation Physics, Medical Physics and Biomedical Engineering, Sahlgrenska University Hospital, Gothenburg SE-413 45, Sweden; Department of Radiation Physics, Institute of Clinical Sciences, Sahlgrenska Academy, University of Gothenburg, Gothenburg SE-413 45 Sweden; Department of Diagnostic Radiation Physics, Medical Physics and Biomedical Engineering, Sahlgrenska University Hospital, Gothenburg SE-413 45, Sweden

## Abstract

This study compares dual-energy computed tomography (DECT) images of a phantom including different material inserts and with additional lateral titanium or stainless steel inserts, simulating bilateral hip prostheses. Dual-source (DS) and fast kV-switching (FKS) DECT with/without metal artefact reduction (MAR) were compared with regards to virtually monoenergetic CT number accuracy and the depiction of different materials. Streak artefacts were observed between the metal inserts that were more severe with steel compared to titanium inserts. The artefact severity and CT number accuracy depended on the photon energy (keV) for both DECT techniques. While MAR generally increased the CT number accuracy and material depiction within the streak artefacts, it sometimes decreased the accuracy outside the streak artefacts for both DS and FKS. FKS depicted the metal inserts more accurately than DS with regards to both CT numbers and external diameter.

## INTRODUCTION

Treatment planning is necessary prior to starting a course of radiotherapy. This is most often based on a computed tomography (CT) examination to obtain the outline of the patient, to delineate target and organs at risk (OAR) volumes, and to allow the absorbed dose distribution to be calculated. Metal implants in the examined body region cause degradation of CT image quality due to photon starvation and beam hardening^(^^1^^,^  ^2^^)^. The geometric depiction of structures and their CT numbers are important in radiotherapy with photons, and especially in radiotherapy with protons.

CT numbers obtained from traditional polychromatic CT scanners are dependent of the detector material, and the mean energy of the photon energy spectrum, which in turn depends on the beam filtration. Thus, different CT numbers can be obtained when examining the same object, using the same nominal tube voltage (kV) with different CT scanners^(^^3^^,^  ^4^^)^.

Dual energy CT (DECT) scanners utilize two X-ray spectra, to produce virtually monoenergetic (VME) CT images, which has been shown to reduce beam hardening artefacts compared to traditional polychromatic CT scanners^(5)^. The reconstruction of a VME image is based on the assumption that the attenuation is a combination of Compton scattering and photoelectric absorption, and that these interaction processes can be described as monotonic functions of the photon energy. Theoretical CT numbers can be calculated for materials and tissues with known densities and elemental compositions, at monoenergetic photon energies, and should therefore be independent of the scanner used. However, different manufacturers use different solutions to produce DECT images, e.g. dual scan, dual source (DS), dual layer detector and fast kV-switching (FKS).

A DS scanner uses two X-ray tube–detector pairs mounted at an angle of approximately 90 degrees, allowing different tube voltages and tube current modulation to be used for each X-ray tube. An additional tin (Sn) filter can be applied to the high-voltage tube to increase the spectral separation of the two X-ray spectra. The monoenergetic images are created in image space from the CT numbers of the original high and low tube voltage images. One disadvantage of the DS technique is that the measured projections must be corrected for cross-scatter from the other tube. Another disadvantage for DS-DECT is the limited field of view (FOV) that can be scanned (350 mm, as one detector must be smaller due to lack of space in the CT gantry). The DS technique is also more sensitive to patient movement than FKS because of the angular separation between the tubes^(6)^.

In the FKS DECT scanner the tube voltage is rapidly switched between a high and low voltage at a frequency of up to 4.8 kHz, allowing interlaced high- and low-energy projections to be registered within microseconds^(7)^. The acquired sinogram raw data can then be analysed with projection-based basis material decomposition. This method converts the two measured attenuation sinograms into water and iodine sinograms. These sinograms can then be reconstructed as water–iodine basis material images or multiplied by the mass attenuation coefficient of each material to create VME CT numbers. In theory, this method eliminates beam hardening artefacts^(^^5^^,^  ^8^^)^. However, artefacts arising from scattering are not removed. Advantages of FKS-DECT compared to DS-DECT are the almost identical projections of the dual X-ray spectra which makes it less sensitive to patient movement, and the larger FOV of 500 mm. Disadvantages of FKS-DECT are the lack of additional spectral filtration of the high-energy spectrum, and possibility of tube current modulation.

Additional manufacturer proprietary metal artefact reduction (MAR) algorithms can be used in the reconstruction process to reduce the artefacts caused by photon starvation^(9)^. This is especially important in images containing objects with high atomic numbers or large quantities of metal such as steel. The ability to correctly define the target and the OAR volumes is a cornerstone in radiotherapy treatment planning. Delineation can be challenging when the patient has metal implants. The use of VME images with MAR has been shown to improve the delineation of objects close to steel implants^(10)^. The CT number accuracy (and precision) is important as any errors introduced to the absorbed dose calculation to the patient will be systematic throughout the therapy course.

In addition to VME images, DECT scanners can also reconstruct images based on the effective atomic number (*Z_eff_*) of materials and tissues. These images can be of interest, for example, in the identification of kidney stones in diagnostic radiology^(11)^, and for determining proton stopping power ratios required for the calculation of the absorbed dose distribution during the planning of proton radiotherapy^(12–14)^.

The aim of this study was to compare two DECT scanners based on different techniques, DS and FKS, from two different manufacturers. The work focused on image properties of interest for radiotherapy treatment planning. The reproduction of VME CT numbers, *Z_eff_* and the geometry of different materials in an electron density phantom were studied under the influence of metal inserts of steel or titanium, and with/without the use of MAR algorithms. The depiction of the metal inserts was also evaluated.

## MATERIALS AND METHODS

### The phantom

An electron density phantom (062 M, CIRS Inc.) made of Plastic Water**®-**LR (Low Range, water equivalent between 15 keV to 8 MeV) with the dimensions of the cross section of a human pelvis, 330 mm × 270 mm × 50 mm, was used in this study. The phantom has several holes into which different materials can be inserted. The tissue surrogate inserts included with the phantom, corresponding to adipose tissue, breast, muscle, liver, trabecular bone and dense bone, were used. An insert of liquid water, as well as additional non-tissue-equivalent material inserts were also used: graphite, polytetrafluoroethylene (PTFE) and air (i.e. no insert).

Bilateral hip prostheses were simulated by in-house developed metal inserts of either stainless steel or titanium. These were 50 mm long and had an inner metal core with a diameter of 10 mm surrounded by 4 mm polyoxymethylene (POM) and an outer metal casing with a thickness of 6 mm, resulting in a total external diameter of 30 mm^(^^10^^,^  ^15^^)^. For comparison, non-metal images were acquired by using bilateral inserts of solid polymethyl methacrylate (PMMA).

The elemental compositions of the phantom and its inserts were obtained from the manufacturer^(16)^. The elemental compositions of PTFE, graphite, liquid water, titanium and POM were taken from the NIST STAR database^(17)^. The mass densities (${\rho}_{material}$) of the tissue surrogate, PTFE and graphite, were obtained by measurements, while the mass densities of titanium and POM were taken from the NIST STAR database^(17)^. The density and elemental composition of 316 L grade stainless steel were obtained from the literature^(18)^. This information, together with the elemental photon mass attenuation data ($\frac{\mu }{\rho }$) from the NIST XCOM database^(19)^, was used to calculate theoretical monoenergetic CT numbers for each material according to equation 1.

(1) \begin{eqnarray*} CT- number\ \left[ HU\right] &&=1000\cdot \left(\frac{\mu_{material}-{\mu}_{water}}{\mu_{water}-{\mu}_{air}}\right)\nonumber\\ &&\approx 1000\cdot \left(\frac{\mu_{material}}{\mu_{water}}-1\right) \end{eqnarray*}

where

(2) \begin{equation*} {\mu}_{material}={\rho}_{material}\cdot{\sum}_i{\omega}_i{\left(\frac{\mu }{\rho}\right)}_i \end{equation*}

and ${\omega}_i$ is the mass fraction of element $i$ in the material.

### DECT scans

The phantom was scanned three times in each DECT scanner with lateral inserts of either steel, titanium or PMMA using clinical scan protocols. To achieve patient-like scattering conditions, Plastic Water™ slabs, 100 mm thick, were added cranially and caudally of the 50 mm thick phantom.

The DS scans were performed with a DS DECT scanner (Somatom Force, Siemens Healthineers, Forchheim, Germany) with tube voltages of 100 kV (tube A) and 150 kV, with additional tin filter (tube B), and with separate tube current modulation for each tube. The paired 100 kV and 150 Sn kV images were used to create VME and *Z_eff_* images in Syngo.Via™ (version VB30A) using the Monoenergetic plus and the Rho/Z applications, respectively.

The FKS scans were performed on a DECT scanner (Revolution CT, GE Healthcare, Waukesha, USA) using FKS scan mode (Gemstone Spectral Imaging™, GSI) with a fixed tube current for both tube voltages (80 and 140 kV). The FKS VME images were reconstructed on the CT console. These images were exported as proprietary GSI data file and sent to the Advantage Workstation Server 3.2™, where *Z_eff_* images were produced using the GSI Volume Viewer™ application (version vxtl_13.0-4.126).

The scanning and reconstruction parameters used are presented in Table 1. VME images were reconstructed at photon energies from 40 to 140 keV in 10 keV steps, and *Z_eff_* images were reconstructed with and without each scanner’s MAR algorithm.

**Table 1:** The scanning and reconstruction parameters used in the two DECT techniques.

	Dual Source	Fast kV-Switching
CT software version	Syngo CTVT VB20A	revo_ct_21b.32
Tube voltage [kV]	100 and 150 Sn	80 and 140
Tube current [mA]	Modulated	435
Rotation time [s]	0.5	1.0
Helical pitch	0.5	0.516
Detector collimation [mm]	38.4 and 38.4	40
Detector size in z-axis [mm]	0.6	0.625
Image slice thickness [mm]	2	2.5
Recon filter kernel	Qr32d	Standard
Iterative reconstruction, (strength)	ADMIRE, (level 3 of 5)	ASIR-V, (60%)
Data collection diameter [mm]	500	500
Field of View [mm]	350	350
Image matrix size	513 × 513	512 × 512
CTDI_vol,32cm_ [mGy]^a^	10.7	45.3

^a^CTDI_vol, 32 cm_, Computed Tomography Dose Index, by volume, in the body phantom (⌀ = 32 cm).

### Image analysis

#### CT numbers

**Figure 1:**
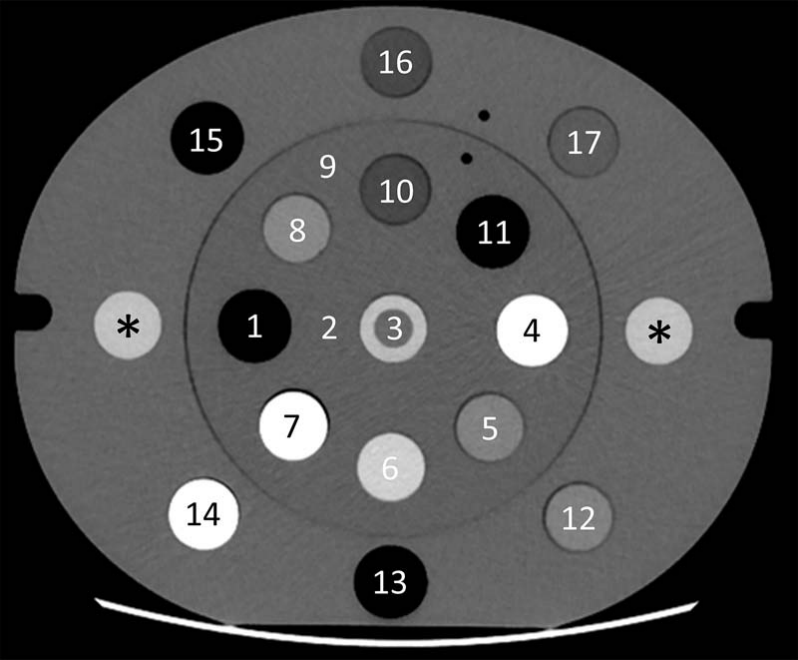
Virtual monoenergetic (110 keV) fast kV-switching DECT image of the electron density phantom with the inserts, where the positions 1–4 (central) and 5–17 (peripheral). The asterisks (^*^) indicate the lateral insert positions where the CT numbers and profiles of the metal inserts were measured. Note that in this image the lateral inserts are of PMMA.

Image analysis was performed in MATLAB™ (version R2019a, Natick, USA) on the central transaxial image of the phantom (Figure 1). The location of the lateral inserts of PMMA, steel or titanium is indicated by asterisks in Figure 1. The mean CT numbers and standard deviation of the inserts and the phantom itself were measured in regions of interest (ROIs) with a 14 mm diameter, except for the metal core inserts, where a 4 mm diameter ROI was used. The mean CT numbers of each material were compared to the calculated theoretical CT numbers. The root-mean-square error (RMSE) of the measured CT numbers was calculated separately for inserts in the central part of the phantom, i.e. in between the metal inserts (Figure 1, ROIs 1–4) and peripheral positions (Figure 1, ROIs 5–17).

#### Effective atomic number, *Z_eff_*

The *Z_eff_* images were evaluated based on the mean *Z_eff_* in the ROI of the graphite insert (at position 7, in Figure 1) which was the only material investigated that was composed of a pure chemical element (Z = 6).

#### Depiction of the metal inserts

The depiction of the metal inserts was evaluated visually by extracting the horizontal and vertical profiles across the inserts in the images obtained at 70, 110 and 140 keV, and comparing them to the known geometry and the theoretical CT numbers. The external diameters were estimated as the widths of the profiles at half the measured CT number in the core ROIs.

#### Depiction of bone and air between metal

The depiction of the dense bone (position 4 in Figure 1) and air (position 1 in Figure 1), both positioned in between the lateral metal inserts, were studied. Delineation of the bone and air structures were performed separately in each image based on CT number thresholding inside a 50 mm diameter circle at half the measured mean CT number in the ROIs for each material. A reference structure, i.e. true dimensions, was defined as a circle of 30 mm in diameter. The thresholded delineations of bone and air were compared to the reference structure based on the area and Dice similarity coefficient (DSC)^(20)^ of two volumes X and Y as defined in equation 3.

(3) \begin{equation*} DSC=\frac{2\cdot \left|X\cap Y\right|}{\left|X\right|+\left|Y\right|} \end{equation*}

The DSC is equal to unity for identical volumes and null for non-overlapping (disjoint) volumes. Due to the difference in pixel dimension of the DS and FKS images, the area of the reference structure was slightly different for the different imaging techniques, i.e. 702.3 mm^2^ for FKS and 704.3 mm^2^ for DS. The difference in area between this reference structure and the true area of a 30 mm circle, i.e. 706.9 mm^2^, can be regarded as within the uncertainty of the structure outline due to the pixel size.

## RESULTS

**Figure 2:**
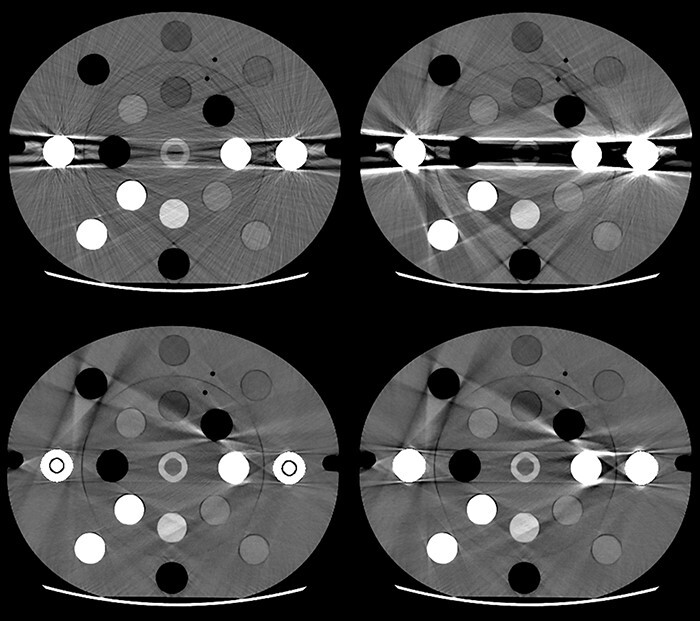
Visual examples of scans illustrating four 110 keV fast kV-switching DECT images of the electron density phantom with titanium (left) and steel (right) inserts. The upper images were reconstructed without metal artefact reduction (MAR), and the lower images with MAR. The images are displayed with a window level/width of 40/400 HU.

Streak artefacts were observed between the lateral metal inserts. There were more artefacts with stainless steel than with titanium in non-MAR images from both DECT techniques, resulting from the higher density of steel. Dense bone and air situated in between the lateral metal inserts were both deformed by the artefacts, even in images with MAR (Figure 2).

### CT numbers

The largest CT number deviations were seen for the materials, except air, placed in between the lateral metal inserts (ROIs 1–4) within the main streak artefacts in the images without MAR (Table 2). The RMSEs in these ROIs were generally lower when using MAR, and lower for DS-MAR compared to FKS-MAR. The VME images reconstructed at 70 keV resulted in large deviations for these materials for both titanium and steel inserts (>486 HU), while the images at 110 keV resulted in large deviations only for steel (>259 HU for steel and <50 HU for titanium). These deviations were similar for both DECT techniques, for all materials within the streak artefacts except for air, where the deviations for FKS resulted in larger deviations compared to DS. MAR improved the results for all the materials with large CT number deviations. The complete data for all energies are presented in the Supplementary Material.

**Table 2:** The mean values and standard deviations of the CT numbers for different materials measured in virtually monoenergetic images obtained with Dual Source (DS), and fast kV-switching (FKS) DECT techniques, and the aforementioned combined by each vendor’s proprietary metal artefact reduction (MAR) algorithm (DS-MAR and FKS-MAR) at (a) 70 keV and (b) 110 keV, together with the theoretically calculated CT numbers. The images were obtained with lateral inserts of PMMA, titanium and stainless steel. The locations of ROI-positions 1–17 are illustrated in Figure 1. The corresponding data for additional energies between 40 and 140 keV are found in tables S1–S11 in the supplementary materials.

a)																						
		Mean CT number (Standard Deviation) [HU] @ 70 keV																				
Material	Position	Theoretical	PMMA				Titanium								Steel							
			DS		FKS		DS		FKS		DS-MAR		FKS-MAR		DS		FKS		DS-MAR		FKS-MAR	
Air	1	**−1000**	−986	(6)	−997	(7)	−1016	(6)	−1195	(55)	−978	(14)	−1002	(10)	−1023	(1)	−1248	(75)	−962	(11)	−999	(15)
Plastic Water-LR	2	**0**	−1	(8)	−4	(7)	−256	(62)	−372	(77)	−10	(13)	−29	(14)	−638	(122)	−486	(100)	−3	(14)	−25	(14)
Liquid water	3	**0**	14	(3)	1	(7)	−267	(108)	−396	(158)	4	(12)	−35	(17)	−665	(225)	−511	(184)	11	(16)	−40	(17)
Dense bone	4	**1618**	1510	(9)	1587	(15)	1299	(45)	1252	(53)	1608	(20)	1549	(35)	939	(58)	1114	(89)	1621	(22)	1531	(55)
Muscle	5	**46**	51	(8)	50	(7)	70	(17)	59	(26)	34	(16)	27	(9)	78	(38)	41	(27)	26	(18)	25	(13)
Trabecular bone	6	**280**	278	(7)	280	(8)	292	(28)	273	(31)	218	(10)	276	(27)	267	(45)	248	(28)	206	(10)	278	(29)
Graphite	7	**462**	460	(13)	466	(12)	466	(30)	431	(38)	383	(54)	449	(22)	345	(26)	379	(23)	358	(59)	442	(22)
Liver	8	**55**	57	(6)	59	(6)	61	(27)	53	(14)	30	(10)	36	(8)	40	(43)	32	(13)	16	(7)	39	(7)
Plastic Water-LR	9	**0**	−3	(6)	−4	(6)	34	(17)	23	(16)	21	(9)	14	(25)	77	(36)	46	(26)	15	(12)	20	(25)
Adipose tissue	10	**−68**	−62	(6)	−61	(5)	−47	(18)	−45	(20)	−56	(12)	−82	(25)	−35	(55)	−27	(16)	−64	(14)	−72	(30)
Lung Inhale	11	**−830**	−818	(7)	−825	(8)	−781	(19)	−762	(19)	−815	(16)	−860	(10)	−664	(56)	−702	(35)	−815	(19)	−864	(10)
Muscle	12	**46**	53	(7)	49	(6)	50	(11)	43	(14)	43	(6)	43	(7)	52	(16)	46	(11)	41	(10)	42	(7)
Air	13	**−1000**	−988	(5)	−996	(6)	−971	(7)	−949	(13)	−968	(6)	−988	(12)	−881	(14)	−902	(12)	−958	(9)	−988	(11)
PTFE	14	**944**	956	(11)	957	(9)	937	(17)	908	(25)	961	(17)	951	(13)	842	(21)	865	(12)	945	(13)	951	(13)
Lung Exhale	15	**−524**	−520	(6)	−524	(5)	−512	(12)	−492	(18)	−503	(13)	−466	(6)	−464	(13)	−467	(12)	−502	(18)	−464	(6)
Adipose tissue	16	**−68**	−58	(6)	−62	(6)	−64	(13)	−67	(11)	−57	(5)	−51	(6)	−76	(8)	−60	(10)	−60	(7)	−51	(7)
Breast	17	**−32**	−22	(5)	−26	(6)	−32	(10)	−22	(13)	−27	(5)	−21	(6)	−49	(10)	−27	(13)	−34	(6)	−23	(6)
Air	1	**−1000**	−986	(6)	−990	(5)	−1016	(6)	−992	(31)	−979	(13)	−990	(8)	−1023	(1)	−1134	(63)	−964	(9)	−988	(10)
Plastic Water-LR	2	**−1**	−10	(7)	−11	(4)	−18	(19)	−51	(24)	−18	(11)	−24	(9)	−314	(74)	−299	(61)	−8	(13)	−20	(9)
Liquid water	3	**0**	8	(3)	3	(5)	4	(19)	−24	(51)	7	(8)	−9	(7)	−315	(132)	−286	(151)	11	(10)	−16	(11)
Dense bone	4	**984**	954	(9)	934	(9)	969	(16)	938	(33)	975	(19)	921	(19)	693	(65)	725	(56)	982	(19)	914	(34)
Muscle	5	**43**	45	(6)	43	(4)	46	(10)	35	(10)	27	(11)	27	(5)	50	(23)	52	(13)	22	(11)	24	(8)
Trabecular bone	6	**175**	170	(7)	165	(5)	175	(9)	167	(10)	133	(7)	155	(14)	191	(21)	159	(14)	126	(8)	157	(16)
Graphite	7	**500**	496	(9)	494	(8)	495	(12)	485	(12)	463	(19)	477	(13)	500	(25)	464	(12)	458	(21)	474	(14)
Liver	8	**52**	55	(5)	51	(4)	51	(9)	44	(9)	29	(8)	26	(5)	43	(33)	44	(9)	20	(8)	31	(4)
Plastic Water-LR	9	**−1**	−7	(5)	−11	(4)	−2	(8)	−9	(10)	14	(9)	2	(22)	3	(13)	−3	(11)	10	(9)	8	(23)
Adipose tissue	10	**−49**	−44	(6)	−47	(3)	−43	(7)	−49	(11)	−39	(8)	−70	(20)	−35	(13)	−37	(7)	−41	(9)	−64	(26)
Lung Inhale	11	**−828**	−818	(5)	−816	(6)	−811	(9)	−812	(10)	−810	(16)	−852	(7)	−770	(31)	−776	(15)	−814	(17)	−862	(8)
Muscle	12	**43**	46	(5)	41	(4)	44	(8)	39	(8)	38	(6)	35	(5)	41	(10)	44	(7)	36	(5)	35	(4)
Air	13	**−1000**	−988	(5)	−991	(4)	−971	(6)	−984	(9)	−968	(6)	−984	(9)	−971	(8)	−964	(8)	−961	(6)	−984	(9)
PTFE	14	**897**	911	(9)	888	(6)	917	(12)	886	(12)	909	(13)	884	(8)	905	(13)	843	(8)	907	(11)	882	(9)
Lung Exhale	15	**−525**	−522	(5)	−519	(4)	−518	(8)	−515	(11)	−504	(10)	−465	(4)	−513	(9)	−496	(7)	−507	(16)	−462	(4)
Adipose tissue	16	**−49**	−42	(4)	−46	(4)	−45	(6)	−49	(6)	−39	(5)	−35	(4)	−49	(10)	−52	(5)	−38	(6)	−36	(5)
Breast	17	**−20**	−10	(6)	−18	(4)	−7	(7)	−17	(7)	−14	(4)	−14	(4)	−5	(7)	−19	(7)	−20	(5)	−17	(4)

**Figure 3:**
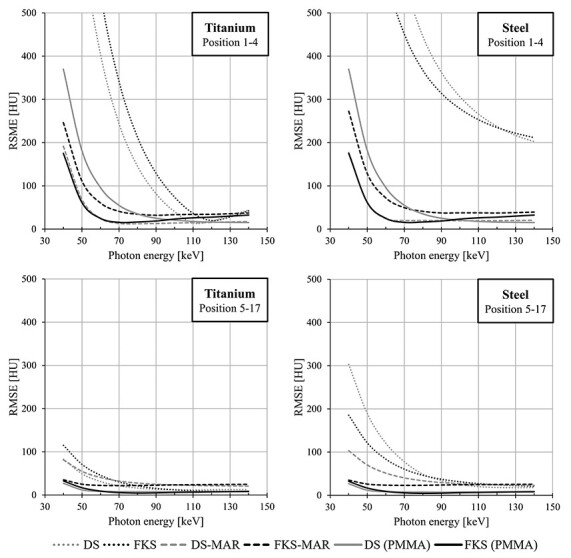
The root mean square error (RMSE) of the CT numbers obtained from scans with titanium (left) or steel (right) inserts, as a function of virtual photon energy level for the central positions 1–4 (top row) and the peripheral positions 5–17 (bottom row) (as defined in Figure 1). The images were acquired with Dual Source (DS) and fast kV-switching (FKS) DECT techniques, and the aforementioned combined by each vendor’s proprietary metal artefact reduction (MAR) algorithm (DS-MAR and FKS-MAR). Corresponding data from images with PMMA inserts are included for comparison.

The overall lowest RMSE for the CT numbers in the ROIs placed in between the lateral metal inserts was found for DS-MAR with a minimum at 80 keV for titanium inserts and at 100 keV for steel inserts (Figure 3). These RMSEs were lower than the corresponding values in the images obtained with PMMA inserts. In the peripheral ROIs, the overall minimal RMSE for the CT numbers in the metal images was found for non-MAR FKS at 140 keV with titanium inserts, and non-MAR DS at 130 keV with steel inserts. The relation in RMSE between different images showed a larger variation with energy for the peripheral ROIs compared to the ROIs in line between the lateral inserts. It should be noted that absolute CT numbers cannot be compared at different energies.

**Figure 4:**
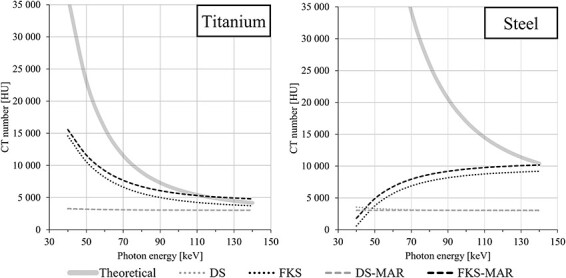
The mean CT numbers measured in 4 mm diameter ROIs in the centre core of the titanium (left) and steel (right) inserts in virtually monoenergetic CT images with photon energies from 40 to 140 keV. The images were acquired with Dual Source (DS) and fast kV-switching (FKS) DECT techniques, and the aforementioned combined by each vendor’s proprietary metal artefact reduction (MAR) algorithm (DS-MAR and FKS-MAR). The theoretical CT numbers of each metal are given for comparison (solid grey line).

For photon energies lower than 110 keV, the measured mean CT number in the ROIs of the metal inserts was always lower than the theoretical CT number for both DECT techniques (Figure 4). The CT number obtained for the metals with DS and DS-MAR was saturated near 3071 HU for high energies, except for steel with DS which reached 3500 HU at the lowest energies. The theoretical CT number of titanium was obtained at 140 keV with FKS, and at 110 keV or higher with FKS-MAR. In the case of steel, the CT numbers obtained with FKS and FKS-MAR increased with increasing energy, which is the opposite relation to the one for the theoretical values where the CT numbers decrease with increasing energy.

### Effective atomic number, *Z_eff_*

In *Z_eff_* images without metal artefacts (i.e. with PMMA inserts), DS was more accurate than FKS in determining the atomic number of graphite (all *Z_eff_* measurements are presented in Table 3). DS also resulted in more accurate *Z_eff_* values than FKS in images with titanium without MAR, but the result was the opposite when using MAR. Both techniques were inaccurate for the non-MAR images with steel inserts. While MAR somewhat improved the results for DS, the most accurate result for images with steel inserts was obtained with FKS-MAR.

**Table 3:** The mean (and standard deviation in parentheses) effective atomic number (*Z*_*eff*_) measured in the graphite insert at position 7 (Figure 1) in *Z*_*eff*_ images obtained with Dual Source (DS) and fast kV-switching (FKS) DECT techniques, and the aforementioned combined by each vendor’s proprietary metal artefact reduction (MAR) algorithm (DS-MAR and FKS-MAR). The images were acquired with lateral inserts of PMMA, titanium or stainless steel. The measurements should be compared to the true atomic number of carbon (Z = 6).

	Effective atomic number (*Z*_*eff*_) of graphite		
Lateral inserts	PMMA	Titanium	Steel
**DS**	6.3 (0.4)	6.3 (1.6)	0.0 (0)
**FKS**	6.7 (0.1)	3.8 (2.1)	1.7 (1.0)
**DS-MAR**	-	2.9 (2.8)	2.2 (2.5)
**FKS-MAR**	-	6.5 (0.3)	6.3 (0.2)

### Depiction of the metal inserts

**Figure 5:**
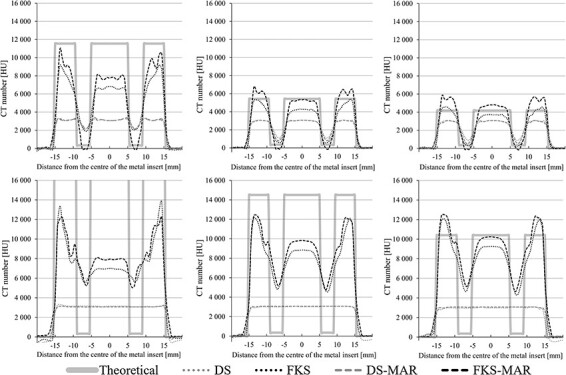
Horizontal CT number profiles for the titanium (top row) and steel (bottom row) inserts at 70 keV (left), 110 keV (middle) and 140 keV (right). The known dimensions of the different materials within the inserts as well as the theoretical CT numbers of titanium, steel, polyoxymethylene and the surrounding Plastic Water materials are shown by the solid grey line for comparison. The images were acquired with Dual Source (DS) and fast kV-switching (FKS) DECT techniques, and the aforementioned combined by each vendor’s proprietary metal artefact reduction (MAR) algorithm (DS-MAR and FKS-MAR). Note that the theoretical CT number of steel at 70 keV is out of scale as the value is 34 287 HU.

The CT number profile analysis revealed that neither DS nor DS-MAR was able to depict the POM surrounding the metal core inside the steel inserts (Figure 5). The measured CT numbers of the POM in the titanium inserts were larger than the theoretical CT number in all cases except for FKS-MAR where it was lower than the theoretical value for all photon energies.

The measured external diameters of the metal inserts were always larger than the actual diameter and in general more accurately depicted with FKS compared to DS (Table 4). The diameters were in general larger in the vertical than in the horizontal direction, except for the diameter for titanium using FKS at 140 keV. MAR was shown to have little influence on the measured diameters.

### Depiction of bone and air between metal

The DSCs for the structures of bone and air positioned in between the lateral inserts were ≥0.97 for all images without metal artefacts (i.e. with PMMA inserts) (Table 5). Therefore, all DSCs ≥0.97 were considered within the range of uncertainties. In the images with titanium inserts, a DSC ≥0.97 was achieved for all energies above 80 keV for both the bone and air structures. The DSC for the bone and air structures in the images with steel was improved when using MAR for both DS and FKS. The results were in all aspects similar for both DS and FKS. The area of the bone structure in the images without metal was always smaller than the reference, while the area of the air structure in the same images was larger. Although, the area differences were always smaller than 2.5%, the largest deviations were observed for low photon energies in images with steel inserts obtained with DS without MAR.

## DISCUSSION

This study is focused on metal artefacts from simulated bilateral hip prostheses, which is a common scenario in the radiotherapy clinic in need of research^(21)^. An advantage with a phantom study is that the same object with known elemental composition and densities in an identical geometry can be used with both scanners. However, the comparison between the two DECT techniques is dependent on the geometry used and the results can be different for other geometries.

If the RMSE is equal for two photon energies, the CT number accuracy in the lower energy image should be considered more accurate as the theoretical CT number difference between tissues is larger at lower energies. MAR improved the CT number accuracy in the region with streak artefacts between the metal inserts for all energies in the case of steel and for energies lower than 110 keV for titanium. This was the case for both DECT techniques studied. However, further away from the metal, i.e. in the peripheral ROIs, MAR could reduce the CT number accuracy. This has previously been reported by others^(^^22^^,^  ^23^^)^. MAR techniques have been developed to correct for visible metal artefacts to improve diagnostic radiology, supposedly at the cost of additional artefacts. The general recommendations from literature and the manufacturers are to study both the MAR and non-MAR images for diagnosis. However, when images are used for radiotherapy treatment planning absorbed dose calculations, the CT number accuracy in the whole image is important for the accuracy of the dose calculation and using multiple images for dose calculations is not straightforward. MAR algorithms should therefore be used with caution in images used for radiotherapy dose calculations. For dental metal implants, MAR has been shown to deteriorate metal geometry such that photon treatment plans were affected^(2)^. Further developments that enable treatment planning based on information from different image reconstructions in combination, i.e. from both MAR and non-MAR images, are desirable. This could be possible, for example if the reading of ‘GSI data files’ created with the FKS technique would be enabled in treatment planning systems.

The depiction of bone and air structures in the region with streak artefacts was improved with MAR due to the reduced artefacts. The delineation of the structures was performed based on CT number thresholding inside a 50 mm diameter circle. Any streak artefact with CT numbers higher (lower) than the threshold level was interpreted as bone (air). A visual structure definition might have given a different result. However, the conditions applied avoided the subjectivity of visual examinations and was the same for both DECT techniques to be compared. Furthermore, an image reconstruction with a smaller FOV centred at the structure might have improved the structure depiction. This is, however, not how these images are used in the clinic, and the 350 mm FOV, used in this study, is smaller than what is often used in clinical practice.

**Table 4:** The measured diameters of the metal inserts (mm) in virtually monoenergetic CT images acquired with Dual Source (DS) and fast kV-switching (FKS) DECT techniques, and the aforementioned combined by each vendors proprietary metal artefact reduction (MAR) algorithm (DS-MAR and FKS-MAR). The metal inserts were thresholded at half the measured mean CT numbers in the 4 mm diameter ROIs in the centre of the metal inserts, for each separate image, at 70, 110 and 140 keV. Thresholding was performed in profiles in the vertical (Ver) and horizontal (Hor) directions in the image. The true metal diameter was 30 mm.

Titanium	70 keV		110 keV		140 keV	
	Ver	Hor	Ver	Hor	Ver	Hor
**DS**	31.8	30.9	31.2	30.7	31.1	30.7
**FKS**	30.7	30.2	30.3	30.1	30.1	31.3
**DS-MAR**	31.6	30.9	31.2	30.7	31.1	30.6
**FKS-MAR**	30.5	30.3	30.4	30.2	30.3	31.6
Steel	70 keV		110 keV		140 keV	
	Ver	Hor	Ver	Hor	Ver	Hor
**DS**	31.6	30.7	32.5	31.2	32.0	31.1
**FKS**	33.0	31.3	30.7	30.3	30.5	30.2
**DS-MAR**	31.4	30.6	31.6	31.3	31.6	31.2
**FKS-MAR**	31.7	31.4	30.7	30.1	30.3	30.0

**Table 5:** The Dice similarity coefficients (DSCs) for the structures of (a) dense bone position 4 in Figure 1 and (c) air position 1 in Figure 1 as well as the area percentage difference for the same bone (b) and air (d) structures positioned in between the lateral inserts, compared to the delineated reference structures in different virtual photon energy levels. The images were acquired with Dual Source (DS) and fast kV-switching (FKS) DECT techniques, and the aforementioned combined by each vendor’s proprietary metal artefact reduction (MAR) algorithm (DS-MAR and FKS-MAR). DSCs ≥0.97 are highlighted.

a)												
		DSC (dense bone)										
Lateral insert	DECT technique	Photon energy [keV]										
		40	50	60	70	80	90	100	110	120	130	140
**PMMA**	**DS**	0.98	0.99	0.99	0.98	0.98	0.98	0.98	0.98	0.98	0.98	0.98
	**FKS**	0.97	0.97	0.97	0.97	0.97	0.97	0.97	0.97	0.97	0.97	0.97
**Titanium**	**DS**	0.89	0.92	0.95	0.96	0.97	0.97	0.97	0.98	0.98	0.98	0.97
	**FKS**	0.86	0.88	0.91	0.95	0.96	0.97	0.97	0.97	0.97	0.97	0.97
	**DS-MAR**	0.97	0.97	0.97	0.97	0.97	0.97	0.97	0.97	0.97	0.97	0.97
	**FKS-MAR**	0.96	0.96	0.96	0.97	0.97	0.97	0.97	0.97	0.97	0.97	0.97
**Steel**	**DS**	0.67	0.73	0.80	0.85	0.87	0.90	0.91	0.93	0.93	0.95	0.95
	**FKS**	0.88	0.89	0.89	0.89	0.88	0.88	0.88	0.87	0.86	0.85	0.85
	**DS-MAR**	0.97	0.97	0.97	0.97	0.97	0.97	0.97	0.97	0.97	0.97	0.97
	**FKS-MAR**	0.96	0.96	0.96	0.96	0.96	0.96	0.96	0.96	0.96	0.96	0.96
b)												
		Area difference (dense bone) [%]										
Lateral insert	DECT technique	Photon energy [keV]										
		40	50	60	70	80	90	100	110	120	130	140
**PMMA**	**DS**	−1.0	−0.8	−0.8	−1.2	−1.4	−1.8	−1.9	−2.2	−2.3	−2.6	−2.7
	**FKS**	−0.7	−0.7	−0.8	−0.8	−0.9	−1.0	−1.0	−1.0	−1.0	−1.0	−1.0
**Titanium**	**DS**	19.5	11.2	4.1	2.1	0.3	−0.7	−1.7	−2.7	−3.1	−3.3	−3.6
	**FKS**	26.1	19.5	12.4	4.8	1.0	0.4	0.0	−0.5	−0.6	−0.8	−0.9
	**DS-MAR**	0.3	0.1	−0.3	−0.6	−1.1	−1.3	−1.8	−2.3	−2.3	−2.3	−2.5
	**FKS-MAR**	3.9	3.1	1.9	1.3	0.5	0.3	−0.1	−0.5	−0.5	−0.7	−0.8
**Steel**	**DS**	60.3	54.0	38.1	26.6	20.2	15.8	11.7	9.5	7.3	4.8	3.7
	**FKS**	16.3	14.1	13.5	13.8	12.5	10.0	9.3	8.1	7.6	7.2	6.8
	**DS-MAR**	0.4	0.0	0.0	−0.3	−0.6	−0.9	−1.0	−1.0	−1.2	−1.3	−1.6
	**FKS-MAR**	3.4	3.4	3.3	3.1	2.9	2.9	3.0	2.9	2.9	2.7	2.7
c)												
		DSC (air)										
Lateral insert	DECT technique	Photon energy [keV]										
		40	50	60	70	80	90	100	110	120	130	140
**PMMA**	**DS**	0.98	0.98	0.98	0.98	0.98	0.98	0.98	0.98	0.98	0.98	0.98
	**FKS**	0.98	0.98	0.98	0.98	0.98	0.98	0.98	0.98	0.98	0.98	0.98
**Titanium**	**DS**	0.77	0.84	0.92	0.97	0.98	0.98	0.98	0.98	0.98	0.98	0.98
	**FKS**	0.77	0.83	0.90	0.94	0.97	0.98	0.98	0.98	0.98	0.98	0.98
	**DS-MAR**	0.97	0.98	0.98	0.98	0.98	0.98	0.98	0.98	0.98	0.98	0.98
	**FKS-MAR**	0.98	0.98	0.98	0.98	0.98	0.98	0.98	0.98	0.98	0.98	0.98
**Steel**	**DS**	0.71	0.76	0.80	0.83	0.89	0.93	0.94	0.95	0.95	0.95	0.95
	**FKS**	0.77	0.83	0.89	0.91	0.92	0.92	0.92	0.93	0.93	0.93	0.93
	**DS-MAR**	0.97	0.97	0.98	0.98	0.98	0.98	0.98	0.98	0.98	0.98	0.98
	**FKS-MAR**	0.98	0.98	0.98	0.98	0.98	0.98	0.98	0.98	0.98	0.98	0.98
d)												
		Area difference (air) [%]										
Lateral insert	DECT technique	Photon energy [keV]										
		40	50	60	70	80	90	100	110	120	130	140
**PMMA**	**DS**	1.0	1.2	1.3	1.1	1.1	1.1	1.0	1.0	1.0	1.0	0.8
	**FKS**	1.3	1.5	1.5	1.9	2.1	2.1	2.1	2.2	2.2	2.2	2.3
**Titanium**	**DS**	31.2	20.2	8.0	0.3	0.1	0.2	0.2	0.1	0.2	0.2	0.3
	**FKS**	14.1	8.8	−1.1	−3.8	−1.4	−0.2	0.5	0.9	1.1	1.1	1.2
	**DS-MAR**	−2.4	−0.7	−0.2	0.4	0.6	0.8	0.9	1.0	1.1	1.1	1.2
	**FKS-MAR**	1.7	2.0	2.1	2.5	2.8	2.9	2.9	2.9	2.9	2.9	2.9
**Steel**	**DS**	24.6	16.6	16.3	15.0	6.8	−0.5	−1.2	−2.0	−1.8	−1.0	−1.2
	**FKS**	6.8	4.8	1.1	−0.1	−1.1	−1.2	−1.4	−0.7	−0.8	−0.9	−0.8
	**DS-MAR**	−3.5	−1.7	−0.6	0.2	0.3	0.5	0.6	0.6	0.7	0.8	0.9
	**FKS-MAR**	2.4	2.5	2.7	2.9	3.3	3.3	3.4	3.4	3.4	3.4	3.4

This study illustrates the need for an extended CT scale to depict metal implants correctly. This is in line with findings from Glide-Hurst et al.^(24)^. Furthermore, for an extended CT scale, the bit depth in the image should preferably be 16-bit to retain the standard CT number resolution^(25)^. The FKS VME images had 16-bit depth and used an extended CT scale. The DS VME images were created as 16-bit images based on the 12-bit paired 100 kV/150 Sn kV images. These paired images could not be reconstructed with an extended CT scale, resulting in the DS VME CT numbers of metal being restricted to values slightly above the standard 12-bit limit of 3071 HU.

The results for the measured *Z_eff_* of graphite were quite different for DS and FKS. One interesting difference is that the use of MAR in the metal images improved the results for FKS but deteriorated the results for DS. The study of *Z_eff_* was limited to only one material, i.e. graphite. The reason is that graphite was the only material in the phantom that was not a mix of different materials and therefore had a distinct theoretical *Z*. For mixed materials the theoretical *Z* needs to be calculated based on theory and there is no consensus in how to perform such calculations. Therefore, the theoretical *Z* of mixed materials is not distinct and includes uncertainties. This study therefore gives limited information on the accuracy of *Z_eff_* images and a more extensive study is needed to reveal the full picture.

The measured diameters of the metal inserts showed that the vertical profiles were in general larger and less accurate than the horizontal profiles. This can be expected as the lateral projections of the scan, which resolves the vertical dimensions, need to penetrate both metal inserts which hampers the geometrical depiction. The fact that the DS CT numbers of metal were saturated near 3500 HU might be the reason for the larger diameters that were measured in DS images compared to FKS. MAR did not change the metal geometry for any of the techniques. Comparisons with our earlier study based on images of the same metal inserts obtained with an earlier model of an FKS scanner from the same manufacturer^(15)^, where the FKS-MAR did not preserve the diameter for steel and titanium inserts, indicate that the technique has been improved over time.

The results show that FKS-MAR can obtain CT numbers of metal within 2.5% for steel and titanium. The FKS CT numbers had an unphysical energy dependence for the steel inserts, i.e. increasing CT number with increasing energy. The unphysical energy dependence of the steel CT numbers was also found for another FKS scanner of an older version from the same manufacturer as the one used in the present study^(15)^. The CT numbers presented in Figure 4 are the mean values of the CT numbers in two central ROIs in the cores of the two metal inserts. The CT numbers in the outer metal shell were in general higher than those measured in the core (see Figure 5). The energy that resulted in the highest CT number accuracy varied between the different metals but also on whether MAR was used or not or where in the phantom the CT number was measured.

The analyses in this study are based on one single scan for each phantom set-up and the variations between different scans are not considered. Furthermore, only one scanning protocol for each DECT scanner has been evaluated. The protocols were selected because they are clinically relevant and were in most aspects similar for the two DECT scanners. The level of iterative reconstruction was similar for the two scanners but the CTDI_vol_ was somewhat different (see Table 1). The image noise was, however, considered to be similar for the two DECT techniques based on the similarities in the measured standard deviations of the CT numbers in the ROIs for the different materials in the non-metal images (see Table 2).

## CONCLUSIONS

The CT number accuracy in positions in between titanium or steel inserts and therefore within streak artefacts was improved by using MAR. For these positions, DS-MAR showed a higher accuracy compared to FKS-MAR for both titanium and steel at all VME levels. For both DS and FKS, the greatest CT number accuracy in the peripheral parts of the phantom, i.e. outside the streak artefacts, was achieved without MAR. In these images, DS had higher accuracy compared to FKS for VME energies <100 keV with titanium inserts and for VME energies ≥100 keV with steel inserts. FKS was shown to depict the metal inserts more accurately than DS with regards to both VME CT numbers and external diameter.

## Supplementary Material

SupplementaryMaterials_rev1_ncab105
