# Supplementary material for: COMPARISON OF METAL ARTEFACTS FOR DIFFERENT DUAL ENERGY CT TECHNIQUES
Source: Radiat Prot Dosimetry. 2021 Aug 4;195(3-4):232–45. doi: 10.1093/rpd/ncab105 (PMC8507444; doi:10.1093/rpd/ncab105)
Supplement: SupplementaryMaterials_rev1_ncab105 [file supplementarymaterials_rev1_ncab105.pdf]

Supplementary material:

Comparison of metal artefacts for different dual energy CT techniques

Pettersson E., Bäck A. and Thilander-Klang A.

Table S1. Theoretical monoenergetic CT numbers of the materials in the electron density phantom. The locations of ROI-positions 1-17 are illustrated in figure 1.

		Theoretical CT numbers [HU]										
		Photon energy [keV]										
**Material**	**Position**	**40**	**50**	**60**	**70**	**80**	**90**	**100**	**110**	**120**	**130**	**140**
Air	**1**	-1000	-1000	-1000	-1000	-1000	-1000	-1000	-1000	-1000	-1000	-1000
Plastic Water-LR	**2**	3	1	0	0	-1	-1	0	-1	-1	-1	-1
Liquid water	**3**	0	0	0	0	0	0	0	0	0	0	0
Dense bone	**4**	4118	2799	2054	1618	1351	1179	1065	984	926	884	852
Muscle	**5**	54	50	48	46	45	44	44	43	43	43	43
Trabecular bone	**6**	705	479	353	280	235	207	189	175	166	159	154
Graphite	**7**	305	390	436	462	478	488	496	500	503	505	507
Liver	**8**	64	60	57	55	54	53	53	52	52	52	52
Plastic Water-LR	**9**	3	1	0	0	-1	-1	0	-1	-1	-1	-1
Adipose	**10**	-143	-102	-80	-68	-60	-55	-51	-49	-47	-46	-45
Lung Inhale	**11**	-839	-834	-832	-830	-830	-829	-829	-828	-828	-828	-828
Muscle	**12**	54	50	48	46	45	44	44	43	43	43	43
Air	**13**	-1000	-1000	-1000	-1000	-1000	-1000	-1000	-1000	-1000	-1000	-1000
PTFE	**14**	1137	1034	978	944	924	912	903	897	892	889	886
Lung Exhale	**15**	-522	-523	-524	-524	-524	-525	-525	-525	-525	-525	-525
Adipose	**16**	-143	-102	-80	-68	-60	-55	-51	-49	-47	-46	-45
Breast	**17**	-80	-54	-40	-32	-27	-24	-21	-20	-19	-18	-17

Supplementary material:

Comparison of metal artefacts for different dual energy CT techniques

Pettersson E., Bäck A. and Thilander-Klang A.

Table S2. The measured monoenergetic CT numbers (mean and standard deviation), in Dual Source (DS) DECT images with lateral inserts of PMMA. The locations of ROI-positions 1-17 are illustrated in figure 1.

DS PMMA

		Mean CT number [HU]										
		Photon energy [keV]										
**Material**	**Position**	**40**	**50**	**60**	**70**	**80**	**90**	**100**	**110**	**120**	**130**	**140**
Air	**1**	-986	-986	-986	-986	-986	-986	-986	-986	-986	-986	-986
Plastic Water-LR	**2**	29	15	5	-1	-4	-7	-9	-10	-11	-12	-12
Liquid water	**3**	32	23	17	14	12	10	9	8	8	8	7
Dense bone	**4**	3380	2437	1863	1510	1283	1132	1028	954	900	860	829
Muscle	**5**	74	63	56	51	49	47	45	45	44	43	43
Trabecular bone	**6**	639	458	347	278	234	205	184	170	160	152	146
Graphite	**7**	339	399	436	460	475	485	492	496	500	503	505
Liver	**8**	66	62	59	57	56	56	56	55	55	55	55
Plastic Water-LR	**9**	9	3	-1	-3	-5	-6	-6	-7	-7	-7	-8
Adipose	**10**	-123	-92	-73	-62	-55	-50	-46	-44	-42	-41	-40
Lung Inhale	**11**	-820	-819	-818	-818	-817	-818	-817	-818	-817	-817	-817
Muscle	**12**	75	64	57	53	50	48	47	46	45	45	44
Air	**13**	-988	-988	-988	-988	-988	-988	-988	-988	-988	-988	-988
PTFE	**14**	1107	1031	985	956	938	926	917	911	907	903	901
Lung Exhale	**15**	-512	-516	-518	-520	-521	-521	-522	-522	-522	-522	-523
Adipose	**16**	-112	-84	-68	-58	-51	-47	-44	-42	-40	-39	-38
Breast	**17**	-65	-43	-30	-22	-17	-14	-11	-10	-8	-7	-7

		Standard deviation [HU]										
		Photon energy [keV]										
**Material**	**Position**	**40**	**50**	**60**	**70**	**80**	**90**	**100**	**110**	**120**	**130**	**140**
Air	**1**	6	7	7	6	6	6	6	6	6	6	6
Plastic Water-LR	**2**	18	14	10	8	6	7	7	7	7	7	7
Liquid water	**3**	18	14	11	8	7	7	7	7	7	7	7
Dense bone	**4**	27	19	13	9	8	8	9	9	9	9	9
Muscle	**5**	17	13	10	8	6	6	6	6	6	6	6
Trabecular bone	**6**	24	17	11	7	6	6	7	7	7	7	8
Graphite	**7**	33	23	17	13	11	10	9	9	8	8	8
Liver	**8**	15	11	8	6	5	5	5	5	5	6	6
Plastic Water-LR	**9**	15	11	8	6	5	5	5	5	5	5	5
Adipose	**10**	13	10	8	6	5	5	6	6	6	6	6
Lung Inhale	**11**	14	11	8	7	6	5	5	5	5	5	5
Muscle	**12**	16	12	9	7	6	5	5	5	5	5	5
Air	**13**	5	5	5	5	5	5	5	5	5	5	5
PTFE	**14**	26	19	14	11	10	10	10	9	9	9	9
Lung Exhale	**15**	11	8	7	6	5	5	5	5	5	5	5
Adipose	**16**	15	11	8	6	5	4	4	4	4	4	4
Breast	**17**	12	9	7	5	5	5	6	6	6	6	6

Supplementary material:
Comparison of metal artefacts for different dual energy CT techniques
Pettersson E., Bäck A. and Thilander-Klang A.

Table S3. The measured monoenergetic CT numbers (mean and standard deviation), in fast kV-switching (FKS) DECT images with lateral inserts of PMMA. The locations of ROI-positions 1-17 are illustrated in figure 1.

FKS PMMA

		Mean CT number [HU]											Standard deviation [HU]										
		Photon energy [keV]											Photon energy [keV]										
**Material**	**Position**	**40**	**50**	**60**	**70**	**80**	**90**	**100**	**110**	**120**	**130**	**140**	**40**	**50**	**60**	**70**	**80**	**90**	**100**	**110**	**120**	**130**	**140**
Air	**1**	-1018	-1007	-1001	-997	-994	-992	-991	-990	-989	-989	-989	14	10	8	7	6	6	5	5	5	5	5
Plastic Water-LR	**2**	18	7	0	-4	-7	-9	-10	-11	-11	-12	-12	15	11	9	7	6	5	5	4	4	4	4
Liquid water	**3**	-5	-1	0	1	2	3	3	3	3	3	3	16	12	9	7	7	6	5	5	5	5	5
Dense bone	**4**	3769	2676	2004	1587	1319	1143	1020	934	873	826	789	34	24	18	15	12	11	10	9	8	8	8
Muscle	**5**	74	62	55	50	47	45	44	43	43	42	42	16	12	9	7	6	5	5	4	4	4	4
Trabecular bone	**6**	665	472	354	280	233	202	180	165	154	146	139	17	12	9	8	6	6	5	5	5	4	4
Graphite	**7**	373	420	449	466	478	485	491	494	497	499	500	25	19	15	12	10	9	8	8	7	7	7
Liver	**8**	84	71	64	59	56	54	52	51	50	50	49	14	10	8	6	5	5	4	4	4	4	3
Plastic Water-LR	**9**	18	7	0	-4	-7	-9	-10	-11	-12	-12	-13	13	9	7	6	5	4	4	4	3	3	3
Adipose	**10**	-109	-85	-70	-61	-55	-51	-49	-47	-45	-44	-44	12	9	7	5	5	4	4	3	3	3	3
Lung Inhale	**11**	-853	-839	-830	-825	-821	-819	-818	-816	-816	-815	-815	16	12	10	8	7	7	6	6	6	6	6
Muscle	**12**	74	61	54	49	46	44	42	41	41	40	40	14	10	8	6	5	5	4	4	4	4	4
Air	**13**	-1013	-1005	-1000	-996	-994	-993	-992	-991	-991	-990	-990	11	8	7	6	5	5	5	4	4	4	4
PTFE	**14**	1188	1073	1001	957	929	910	897	888	882	877	873	21	15	11	9	8	7	6	6	6	5	5
Lung Exhale	**15**	-538	-531	-526	-524	-522	-521	-520	-519	-519	-519	-518	11	8	6	5	5	4	4	4	4	3	3
Adipose	**16**	-116	-89	-72	-62	-55	-51	-48	-46	-44	-43	-42	14	10	8	6	5	5	4	4	4	4	3
Breast	**17**	-52	-39	-31	-26	-23	-21	-19	-18	-17	-17	-16	12	9	7	6	5	4	4	4	3	3	3

Supplementary material:
Comparison of metal artefacts for different dual energy CT techniques
Pettersson E., Bäck A. and Thilander-Klang A.

Table S4. The measured monoenergetic CT numbers (mean and standard deviation), in Dual Source (DS) DECT images with lateral inserts of titanium. The locations of ROI-positions 1-17 are illustrated in figure 1.

DS Titanium

		**Mean CT number [HU]**										
		**Photon energy [keV]**										
**Material**	**Position**	**40**	**50**	**60**	**70**	**80**	**90**	**100**	**110**	**120**	**130**	**140**
Air	**1**	-1016	-1016	-1016	-1016	-1016	-1016	-1016	-1016	-1016	-1016	-1016
Plastic Water-LR	**2**	-852	-651	-406	-256	-159	-95	-50	-18	5	22	35
Liquid water	**3**	-832	-648	-439	-267	-156	-83	-32	4	31	51	66
Dense bone	**4**	2409	1849	1509	1299	1164	1074	1013	969	937	913	894
Muscle	**5**	147	108	84	70	60	53	49	46	43	42	40
Trabecular bone	**6**	683	486	366	292	245	212	190	175	163	155	148
Graphite	**7**	370	418	448	466	478	486	491	495	497	499	501
Liver	**8**	86	73	65	61	57	54	52	51	50	49	49
Plastic Water-LR	**9**	152	92	56	34	19	10	3	-2	-6	-9	-10
Adipose	**10**	-58	-52	-49	-47	-45	-44	-44	-43	-43	-42	-42
Lung Inhale	**11**	-685	-734	-763	-781	-793	-802	-807	-811	-814	-817	-818
Muscle	**12**	66	57	53	50	48	46	45	44	44	43	43
Air	**13**	-970	-971	-971	-971	-971	-971	-971	-971	-971	-971	-971
PTFE	**14**	1001	969	949	937	929	924	920	917	915	914	913
Lung Exhale	**15**	-489	-500	-507	-512	-514	-516	-517	-518	-518	-519	-519
Adipose	**16**	-127	-95	-76	-64	-56	-51	-48	-45	-44	-42	-41
Breast	**17**	-118	-75	-48	-32	-22	-15	-10	-7	-4	-2	-1

		**Standard deviation [HU]**										
		**Photon energy [keV]**										
**Material**	**Position**	**40**	**50**	**60**	**70**	**80**	**90**	**100**	**110**	**120**	**130**	**140**
Air	**1**	6	6	6	6	6	6	6	6	6	6	6
Plastic Water-LR	**2**	215	158	99	62	40	27	21	19	20	21	23
Liquid water	**3**	158	211	167	108	71	44	27	19	18	20	23
Dense bone	**4**	176	109	69	45	30	22	18	16	16	17	18
Muscle	**5**	51	33	23	17	13	11	10	10	10	10	10
Trabecular bone	**6**	111	69	44	28	19	12	9	9	9	10	11
Graphite	**7**	108	68	44	30	21	15	12	12	11	12	12
Liver	**8**	89	57	38	27	19	14	11	9	8	8	8
Plastic Water-LR	**9**	55	35	24	17	12	10	9	8	8	8	8
Adipose	**10**	63	40	26	18	12	9	8	7	7	8	8
Lung Inhale	**11**	59	38	26	19	15	12	10	9	9	8	8
Muscle	**12**	31	20	14	11	8	8	8	8	8	8	8
Air	**13**	7	7	7	7	6	6	6	6	6	6	6
PTFE	**14**	54	35	23	17	13	12	12	12	13	13	13
Lung Exhale	**15**	33	22	16	12	10	9	8	8	8	8	8
Adipose	**16**	35	23	16	13	9	7	7	6	6	6	6
Breast	**17**	27	18	12	10	7	6	6	7	7	7	7

Supplementary material:
Comparison of metal artefacts for different dual energy CT techniques
Pettersson E., Bäck A. and Thilander-Klang A.

Table S5. The measured monoenergetic CT numbers (mean and standard deviation), in fast kV-switching (FKS) DECT images with lateral inserts of titanium. The locations of ROI-positions 1-17 are illustrated in figure 1.

FKS Titanium

		Mean CT number [HU]											Standard deviation [HU]										
		Photon energy [keV]											Photon energy [keV]										
**Material**	**Position**	**40**	**50**	**60**	**70**	**80**	**90**	**100**	**110**	**120**	**130**	**140**	**40**	**50**	**60**	**70**	**80**	**90**	**100**	**110**	**120**	**130**	**140**
Air	**1**	-1824	-1532	-1324	-1195	-1112	-1058	-1019	-992	-973	-959	-947	102	99	72	55	45	38	34	31	29	28	26
Plastic Water-LR	**2**	-1444	-907	-577	-372	-241	-154	-94	-51	-21	2	20	271	174	114	77	54	39	30	24	20	18	17
Liquid water	**3**	-1343	-1023	-632	-396	-243	-142	-73	-24	11	38	60	289	343	227	158	113	84	64	51	41	34	29
Dense bone	**4**	2299	1774	1452	1252	1123	1038	980	938	908	886	868	138	95	69	53	44	38	35	33	31	30	29
Muscle	**5**	139	99	74	59	49	43	39	35	33	31	30	93	59	39	26	19	14	11	10	9	9	9
Trabecular bone	**6**	627	450	341	273	230	201	181	167	157	150	144	107	69	45	31	22	16	13	10	9	8	7
Graphite	**7**	251	341	397	431	453	468	478	485	490	494	497	131	84	55	38	27	20	15	12	11	10	9
Liver	**8**	83	68	59	53	49	47	45	44	43	42	42	33	23	17	14	12	10	9	9	8	8	8
Plastic Water-LR	**9**	127	75	43	23	10	1	-5	-9	-12	-14	-16	36	26	19	16	13	12	11	10	9	9	8
Adipose	**10**	-29	-37	-42	-45	-47	-48	-49	-49	-50	-50	-50	51	35	26	20	16	14	12	11	10	10	9
Lung Inhale	**11**	-592	-677	-729	-762	-782	-796	-805	-812	-817	-821	-824	51	35	25	19	15	13	11	10	10	9	9
Muscle	**12**	56	49	45	43	41	40	39	39	38	38	38	35	25	18	14	12	10	9	8	8	7	7
Air	**13**	-832	-891	-927	-949	-964	-973	-980	-984	-988	-990	-992	25	19	15	13	11	10	9	9	8	8	8
PTFE	**14**	980	944	922	908	899	893	889	886	884	883	881	68	46	33	25	19	16	14	12	11	11	10
Lung Exhale	**15**	-419	-456	-478	-492	-502	-508	-512	-515	-517	-518	-520	40	29	22	18	15	13	12	11	11	10	10
Adipose	**16**	-128	-98	-79	-67	-60	-55	-52	-49	-47	-46	-45	26	19	14	11	9	8	7	6	6	6	5
Breast	**17**	-42	-32	-26	-22	-20	-19	-18	-17	-16	-16	-16	34	23	17	13	10	9	8	7	7	6	6

Supplementary material:
Comparison of metal artefacts for different dual energy CT techniques
Pettersson E., Bäck A. and Thilander-Klang A.

Table S6. The measured monoenergetic CT numbers (mean and standard deviation), in Dual Source (DS) DECT images (with metal artefact reduction, MAR) with lateral inserts of titanium. The locations of ROI-positions 1-17 are illustrated in figure 1.

DS-MAR Titanium

		Mean CT number [HU]										
		Photon energy [keV]										
**Material**	**Position**	**40**	**50**	**60**	**70**	**80**	**90**	**100**	**110**	**120**	**130**	**140**
Air	**1**	-976	-977	-978	-978	-979	-979	-979	-979	-979	-979	-979
Plastic Water-LR	**2**	11	0	-6	-10	-13	-15	-17	-18	-19	-19	-20
Liquid water	**3**	-4	1	3	4	5	6	6	7	7	7	7
Dense bone	**4**	3736	2663	2011	1608	1350	1178	1059	975	913	867	832
Muscle	**5**	59	46	38	34	31	29	28	27	26	26	25
Trabecular bone	**6**	503	359	272	218	183	160	144	133	125	118	114
Graphite	**7**	110	248	331	383	416	438	452	463	471	476	481
Liver	**8**	33	31	31	30	30	29	29	29	28	28	28
Plastic Water-LR	**9**	48	34	26	21	18	16	15	14	13	13	12
Adipose	**10**	-108	-81	-65	-56	-49	-44	-41	-39	-37	-36	-35
Lung Inhale	**11**	-833	-824	-819	-815	-813	-812	-811	-810	-810	-809	-809
Muscle	**12**	60	51	46	43	41	39	39	38	37	37	37
Air	**13**	-967	-967	-968	-968	-968	-968	-968	-968	-968	-968	-968
PTFE	**14**	1131	1045	993	961	940	926	916	909	904	900	897
Lung Exhale	**15**	-502	-502	-502	-503	-504	-504	-504	-504	-504	-504	-504
Adipose	**16**	-116	-86	-68	-57	-49	-44	-41	-39	-37	-36	-35
Breast	**17**	-69	-48	-35	-27	-22	-18	-16	-14	-13	-12	-11

		Standard deviation [HU]										
		Photon energy [keV]										
**Material**	**Position**	**40**	**50**	**60**	**70**	**80**	**90**	**100**	**110**	**120**	**130**	**140**
Air	**1**	22	18	15	14	13	13	13	13	12	12	12
Plastic Water-LR	**2**	27	20	16	13	12	12	12	11	11	11	11
Liquid water	**3**	28	20	15	12	9	8	8	8	8	8	8
Dense bone	**4**	37	27	22	20	19	19	19	19	19	19	19
Muscle	**5**	39	27	20	16	14	12	12	11	11	11	10
Trabecular bone	**6**	30	20	14	10	8	7	7	7	7	7	7
Graphite	**7**	179	116	77	54	39	29	23	19	16	14	13
Liver	**8**	25	17	13	10	8	8	8	8	7	7	7
Plastic Water-LR	**9**	21	15	11	9	9	9	9	9	9	9	9
Adipose	**10**	29	21	15	12	10	9	8	8	8	8	8
Lung Inhale	**11**	22	19	17	16	16	16	16	16	16	16	15
Muscle	**12**	17	12	9	6	6	6	6	6	6	6	7
Air	**13**	8	7	7	6	6	6	6	6	6	6	6
PTFE	**14**	44	30	22	17	14	14	13	13	13	13	13
Lung Exhale	**15**	25	18	14	13	11	10	10	10	10	10	10
Adipose	**16**	12	8	6	5	4	4	5	5	5	6	6
Breast	**17**	13	9	6	5	4	4	4	4	5	5	5

Supplementary material:
Comparison of metal artefacts for different dual energy CT techniques
Pettersson E., Bäck A. and Thilander-Klang A.

Table S7. The measured monoenergetic CT numbers (mean and standard deviation), in fast kV-switching (FKS) DECT images (with metal artefact reduction, MAR) with lateral inserts of titanium. The locations of ROI-positions 1-17 are illustrated in figure 1.

FKS-MAR Titanium

		Mean CT number [HU]											Standard deviation [HU]										
		Photon energy [keV]											Photon energy [keV]										
**Material**	**Position**	**40**	**50**	**60**	**70**	**80**	**90**	**100**	**110**	**120**	**130**	**140**	**40**	**50**	**60**	**70**	**80**	**90**	**100**	**110**	**120**	**130**	**140**
Air	**1**	-1041	-1022	-1009	-1002	-997	-994	-992	-990	-989	-988	-987	20	15	12	10	9	9	8	8	7	7	7
Plastic Water-LR	**2**	-47	-38	-33	-29	-27	-25	-24	-24	-23	-23	-22	32	23	17	14	12	10	9	9	8	8	7
Liquid water	**3**	-123	-80	-52	-35	-24	-17	-12	-9	-7	-5	-3	50	32	23	17	13	10	8	7	7	6	6
Dense bone	**4**	3646	2596	1950	1549	1292	1123	1005	921	862	817	782	92	63	46	35	28	24	21	19	17	17	16
Muscle	**5**	28	28	27	27	27	27	27	27	27	27	27	24	17	12	9	8	7	6	5	5	5	5
Trabecular bone	**6**	683	479	354	276	226	194	171	155	143	134	128	67	46	34	27	22	18	16	14	13	12	12
Graphite	**7**	358	403	432	449	460	468	473	477	479	481	482	50	36	27	22	18	16	14	13	13	12	12
Liver	**8**	66	51	41	36	32	29	28	26	26	25	24	19	13	10	8	7	6	5	5	5	4	4
Plastic Water-LR	**9**	54	34	22	14	9	6	4	2	1	1	0	34	29	26	25	24	23	23	22	22	22	22
Adipose	**10**	-121	-101	-89	-82	-77	-74	-72	-70	-69	-68	-68	41	33	28	25	23	22	21	20	20	19	19
Lung Inhale	**11**	-885	-872	-864	-860	-857	-855	-853	-852	-851	-851	-851	23	17	13	10	9	8	7	7	7	7	7
Muscle	**12**	70	56	48	43	40	38	36	35	34	34	33	16	11	9	7	6	5	5	5	4	4	4
Air	**13**	-1002	-995	-991	-988	-986	-985	-984	-984	-984	-983	-983	23	17	14	12	11	10	9	9	9	8	8
PTFE	**14**	1173	1062	993	951	924	906	893	884	878	873	870	30	22	16	13	11	10	9	8	8	8	7
Lung Exhale	**15**	-473	-470	-468	-466	-466	-465	-465	-465	-464	-464	-464	12	9	7	6	5	4	4	4	4	4	4
Adipose	**16**	-105	-78	-61	-51	-45	-41	-38	-35	-34	-33	-32	13	9	7	6	5	5	4	4	4	4	4
Breast	**17**	-42	-31	-25	-21	-18	-16	-15	-14	-14	-13	-13	14	10	7	6	5	5	4	4	4	4	4

Supplementary material:
Comparison of metal artefacts for different dual energy CT techniques
Pettersson E., Bäck A. and Thilander-Klang A.

Table S8. The measured monoenergetic CT numbers (mean and standard deviation), in Dual Source (DS) DECT images with lateral steel inserts. The locations of ROI-positions 1-17 are illustrated in figure 1.

DS Steel

		Mean CT number [HU]											Standard deviation [HU]										
		Photon energy [keV]											Photon energy [keV]										
**Material**	**Position**	**40**	**50**	**60**	**70**	**80**	**90**	**100**	**110**	**120**	**130**	**140**	**40**	**50**	**60**	**70**	**80**	**90**	**100**	**110**	**120**	**130**	**140**
Air	**1**	-1023	-1023	-1023	-1023	-1023	-1023	-1023	-1023	-1023	-1023	-1023	1	1	1	1	1	1	1	1	1	1	1
Plastic Water-LR	**2**	-906	-808	-834	-638	-508	-419	-358	-314	-283	-259	-243	96	166	163	122	97	85	78	74	70	68	69
Liquid water	**3**	-936	-841	-740	-665	-522	-428	-362	-315	-282	-256	-238	86	150	193	225	186	161	144	132	124	118	115
Dense bone	**4**	1744	1338	1091	939	838	771	726	693	668	651	633	340	196	108	58	44	47	56	65	72	77	82
Muscle	**5**	183	129	97	78	67	59	54	50	47	45	42	120	78	53	38	29	25	23	23	23	23	24
Trabecular bone	**6**	532	396	316	267	236	215	201	191	183	178	173	124	84	60	45	34	28	24	22	19	18	18
Graphite	**7**	-168	87	245	345	416	454	481	500	514	524	532	55	39	30	26	25	25	25	25	25	25	24
Liver	**8**	27	33	38	40	41	42	42	43	43	43	45	85	62	50	43	38	35	34	33	32	32	33
Plastic Water-LR	**9**	316	196	122	77	48	27	13	3	-5	-10	-13	141	88	56	36	24	16	13	13	13	14	15
Adipose	**10**	-29	-32	-34	-35	-35	-35	-35	-35	-35	-35	-35	189	121	80	55	39	26	18	13	10	9	9
Lung Inhale	**11**	-302	-482	-594	-664	-713	-739	-757	-770	-779	-786	-790	135	96	71	56	44	38	34	31	29	27	27
Muscle	**12**	82	67	58	52	47	44	42	41	40	39	39	51	34	23	16	13	11	10	10	9	9	10
Air	**13**	-567	-724	-821	-881	-923	-945	-960	-971	-979	-985	-989	44	29	20	14	12	10	8	8	7	7	7
PTFE	**14**	621	730	799	842	875	888	898	905	910	914	915	60	40	28	21	16	14	13	13	13	13	15
Lung Exhale	**15**	-288	-377	-431	-464	-486	-498	-507	-513	-517	-520	-523	28	20	15	13	10	9	9	9	9	9	9
Adipose	**16**	-160	-118	-92	-76	-64	-57	-52	-49	-46	-44	-43	25	16	11	8	8	9	10	10	11	11	12
Breast	**17**	-187	-118	-76	-49	-30	-19	-10	-5	0	3	5	37	23	14	10	8	7	7	7	7	7	8

Supplementary material:

Comparison of metal artefacts for different dual energy CT techniques

Pettersson E., Bäck A. and Thilander-Klang A.

Table S9. The measured monoenergetic CT numbers (mean and standard deviation), in fast kV-switching (FKS) DECT images with lateral steel inserts. The locations of ROI-positions 1-17 are illustrated in figure 1.

FKS Steel

		Mean CT number [HU]											Standard deviation [HU]										
		Photon energy [keV]											Photon energy [keV]										
**Material**	**Position**	**40**	**50**	**60**	**70**	**80**	**90**	**100**	**110**	**120**	**130**	**140**	**40**	**50**	**60**	**70**	**80**	**90**	**100**	**110**	**120**	**130**	**140**
Air	**1**	-1628	-1438	-1321	-1248	-1201	-1170	-1149	-1134	-1123	-1115	-1109	126	100	84	75	70	67	65	63	62	62	61
Plastic Water-LR	**2**	-1109	-797	-605	-486	-409	-359	-324	-299	-282	-268	-258	238	168	126	100	83	73	66	61	57	55	53
Liquid water	**3**	-1262	-887	-656	-511	-418	-358	-316	-286	-265	-249	-236	293	238	204	184	170	161	155	151	148	146	144
Dense bone	**4**	2411	1761	1362	1114	954	850	777	725	689	661	639	206	147	111	89	76	67	60	56	53	51	49
Muscle	**5**	4	22	34	41	46	49	51	52	53	54	55	74	50	36	27	21	17	15	13	12	11	10
Trabecular bone	**6**	549	398	306	248	212	187	170	159	150	144	139	77	52	37	28	22	18	15	14	12	11	11
Graphite	**7**	92	236	324	379	414	437	453	464	473	479	484	62	42	30	23	18	16	14	12	11	11	10
Liver	**8**	-8	12	24	32	37	40	43	44	45	46	47	38	25	18	13	11	10	9	9	9	8	8
Plastic Water-LR	**9**	211	128	77	46	26	12	3	-3	-8	-12	-14	79	52	36	26	20	16	13	11	10	9	8
Adipose	**10**	5	-12	-21	-27	-32	-34	-36	-37	-38	-39	-39	46	31	22	16	12	10	8	7	6	6	6
Lung Inhale	**11**	-456	-579	-655	-702	-733	-753	-767	-776	-783	-788	-792	104	69	48	35	27	21	17	15	13	12	11
Muscle	**12**	56	51	48	46	45	45	44	44	43	43	43	24	17	13	11	9	8	8	7	7	7	7
Air	**13**	-698	-800	-863	-902	-927	-944	-956	-964	-970	-974	-977	27	20	15	12	10	9	8	8	7	7	7
PTFE	**14**	937	901	879	865	856	850	846	843	841	840	838	27	20	15	12	10	9	8	8	7	7	7
Lung Exhale	**15**	-368	-417	-448	-467	-479	-487	-492	-496	-499	-501	-503	28	20	15	12	10	9	8	7	7	6	6
Adipose	**16**	-90	-75	-66	-60	-57	-54	-53	-52	-51	-50	-50	26	18	13	10	8	6	6	5	5	5	5
Breast	**17**	-54	-41	-32	-27	-23	-21	-20	-19	-18	-17	-17	37	25	18	13	10	9	8	7	6	6	6

Supplementary material:
Comparison of metal artefacts for different dual energy CT techniques
Pettersson E., Bäck A. and Thilander-Klang A.

Table S10. The measured monoenergetic CT numbers (mean and standard deviation), in Dual Source (DS) DECT images (with metal artefact reduction, MAR) with lateral inserts of steel. The locations of ROI-positions 1-17 are illustrated in figure 1.

DS-MAR Steel

		Mean CT number [HU]											Standard deviation [HU]										
		Photon energy [keV]											Photon energy [keV]										
**Material**	**Position**	**40**	**50**	**60**	**70**	**80**	**90**	**100**	**110**	**120**	**130**	**140**	**40**	**50**	**60**	**70**	**80**	**90**	**100**	**110**	**120**	**130**	**140**
Air	**1**	-957	-960	-961	-962	-963	-963	-963	-964	-964	-964	-964	19	15	13	11	10	10	9	9	9	9	9
Plastic Water-LR	**2**	18	7	1	-3	-4	-6	-7	-8	-8	-9	-9	24	19	16	14	14	14	13	13	13	13	13
Liquid water	**3**	15	13	12	11	11	11	11	11	11	11	11	35	26	20	16	13	11	10	10	10	9	9
Dense bone	**4**	3767	2685	2027	1621	1361	1187	1067	982	919	873	838	56	38	28	22	19	19	19	19	18	18	18
Muscle	**5**	44	35	30	26	25	23	23	22	22	22	21	44	31	23	18	15	13	12	11	10	10	10
Trabecular bone	**6**	475	339	257	206	173	151	136	126	118	112	108	29	19	14	10	8	7	8	8	8	8	8
Graphite	**7**	33	197	297	358	397	425	444	458	467	475	480	189	123	83	59	43	32	25	21	18	15	14
Liver	**8**	-1	8	13	16	18	19	19	20	20	20	20	17	12	9	7	7	7	8	8	9	9	9
Plastic Water-LR	**9**	32	24	19	15	13	12	11	10	10	10	9	25	18	14	12	10	9	9	9	9	9	9
Adipose	**10**	-141	-102	-78	-64	-55	-48	-44	-41	-39	-37	-36	33	23	17	14	12	10	9	9	9	9	9
Lung Inhale	**11**	-822	-819	-817	-815	-815	-815	-814	-814	-814	-814	-814	24	21	20	19	18	17	17	17	17	17	17
Muscle	**12**	57	49	44	41	39	37	36	36	35	35	34	24	17	12	10	7	6	5	5	5	5	5
Air	**13**	-950	-954	-957	-958	-960	-960	-961	-961	-962	-962	-962	23	16	11	9	7	6	6	6	5	5	5
PTFE	**14**	1087	1015	972	945	928	919	912	907	903	901	898	38	25	18	13	11	11	11	11	12	12	12
Lung Exhale	**15**	-486	-494	-498	-502	-504	-505	-506	-507	-508	-508	-508	36	26	21	18	16	15	15	16	16	16	16
Adipose	**16**	-127	-93	-72	-60	-51	-45	-41	-38	-36	-35	-34	16	11	8	7	6	6	6	6	6	6	6
Breast	**17**	-79	-56	-42	-34	-28	-24	-22	-20	-19	-17	-17	14	10	7	6	5	5	5	5	5	5	5

Supplementary material:
Comparison of metal artefacts for different dual energy CT techniques
Pettersson E., Bäck A. and Thilander-Klang A.

Table S11. The measured monoenergetic CT numbers (mean and standard deviation), in fast kV-switching (FKS) DECT images (with metal artefact reduction, MAR) with lateral inserts of steel. The locations of ROI-positions 1-17 are illustrated in figure 1.

FKS-MAR Steel

		**Mean CT number [HU]**											**Standard deviation [HU]**										
		**Photon energy [keV]**											**Photon energy [keV]**										
**Material**	**Position**	**40**	**50**	**60**	**70**	**80**	**90**	**100**	**110**	**120**	**130**	**140**	**40**	**50**	**60**	**70**	**80**	**90**	**100**	**110**	**120**	**130**	**140**
Air	**1**	-1033	-1016	-1005	-999	-995	-992	-990	-988	-987	-987	-986	30	22	18	15	13	12	11	10	10	10	9
Plastic Water-LR	**2**	-39	-32	-27	-25	-23	-22	-21	-20	-20	-20	-19	31	23	17	14	12	11	10	9	8	8	8
Liquid water	**3**	-119	-80	-55	-40	-30	-24	-19	-16	-14	-13	-11	39	28	22	17	15	13	12	11	11	10	10
Dense bone	**4**	3589	2558	1924	1531	1278	1112	996	914	857	812	778	129	92	69	55	46	40	36	34	32	30	29
Muscle	**5**	27	26	25	25	25	25	24	24	24	24	24	29	21	16	13	11	9	8	8	7	7	7
Trabecular bone	**6**	680	479	355	278	228	196	173	157	146	137	130	70	49	36	29	24	20	18	16	15	14	14
Graphite	**7**	336	389	422	442	455	464	470	474	477	479	481	46	34	27	22	19	17	15	14	14	13	13
Liver	**8**	66	52	44	39	35	33	32	31	30	29	29	18	12	9	7	6	5	5	4	4	4	4
Plastic Water-LR	**9**	60	40	28	20	15	12	9	8	7	6	5	32	28	26	25	24	23	23	23	23	22	22
Adipose	**10**	-100	-86	-77	-72	-69	-67	-65	-64	-63	-63	-62	46	38	33	30	28	27	26	26	25	25	25
Lung Inhale	**11**	-873	-868	-866	-864	-863	-863	-862	-862	-861	-861	-861	21	15	12	10	9	9	8	8	8	8	7
Muscle	**12**	66	54	47	42	39	37	36	35	34	34	33	14	10	8	7	6	5	5	4	4	4	4
Air	**13**	-1000	-994	-991	-988	-987	-986	-985	-984	-984	-984	-984	21	16	13	11	10	10	9	9	8	8	8
PTFE	**14**	1178	1064	994	951	923	904	891	882	876	871	867	29	21	16	13	11	10	9	9	8	8	8
Lung Exhale	**15**	-471	-467	-465	-464	-463	-462	-462	-462	-461	-461	-461	12	9	7	6	5	4	4	4	4	4	3
Adipose	**16**	-102	-76	-61	-51	-45	-41	-38	-36	-34	-33	-32	14	10	8	7	6	5	5	5	4	4	4
Breast	**17**	-44	-34	-27	-23	-20	-19	-17	-17	-16	-16	-15	13	9	7	6	5	4	4	4	3	3	3
